# Late Quaternary climate change explains soil fungal community composition rather than fungal richness in forest ecosystems

**DOI:** 10.1002/ece3.5247

**Published:** 2019-05-09

**Authors:** Niu‐Niu Ji, Cheng Gao, Brody Sandel, Yong Zheng, Liang Chen, Bin‐Wei Wu, Xing‐Chun Li, Yong‐Long Wang, Peng‐Peng Lü, Xiang Sun, Liang‐Dong Guo

**Affiliations:** ^1^ State Key Laboratory of Mycology, Institute of Microbiology Chinese Academy of Sciences Beijing China; ^2^ College of Life Sciences University of Chinese Academy of Sciences Beijing China; ^3^ Department of Biology Santa Clara University Santa Clara California

**Keywords:** contemporary environmental factors, forest ecosystems, Illumina MiSeq sequencing, late Quaternary climate change, soil fungal community

## Abstract

The dramatic climate fluctuations of the late Quaternary have influenced the diversity and composition of macroorganism communities, but how they structure belowground microbial communities is less well known. Fungi constitute an important component of soil microorganism communities. They play an important role in biodiversity maintenance, community assembly, and ecosystem functioning, and differ from many macroorganisms in many traits. Here, we examined soil fungal communities in Chinese temperate, subtropical, and tropic forests using Illumina MiSeq sequencing of the fungal ITS1 region. The relative effect of late Quaternary climate change and contemporary environment (plant, soil, current climate, and geographic distance) on the soil fungal community was analyzed. The richness of the total fungal community, along with saprotrophic, ectomycorrhizal (EM), and pathogenic fungal communities, was influenced primarily by the contemporary environment (plant and/or soil) but not by late Quaternary climate change. Late Quaternary climate change acted in concert with the contemporary environment to shape total, saprotrophic, EM, and pathogenic fungal community compositions and with a stronger effect in temperate forest than in tropic–subtropical forest ecosystems. Some contemporary environmental factors influencing total, saprotrophic, EM, and pathogenic fungal communities in temperate and tropic–subtropical forests were different. We demonstrate that late Quaternary climate change can help to explain current soil fungal community composition and argue that climatic legacies can help to predict soil fungal responses to climate change.

## 1 INTRODUCTION

Earth's past climate changes have played a key role in shaping the evolution and distribution of macroorganisms (Svenning, Eiserhardt, Normand, Ordonez, & Sandel, 2015). During the late Quaternary (the last 2.6 Myr), global temperature oscillated dramatically between cold glacial and warm interglacial periods (Ruddiman, 2014), with major consequences for the diversity and composition of macroorganism communities (Dobrovolski, Melo, Cassemiro, & Diniz‐Filho, 2012; Normand et al., 2011; Svenning et al., 2015; Svenning & Skov, 2007). Late Quaternary climate change has influenced current biodiversity patterns by driving changes in migration, extinction, and speciation rates (Dynesius & Jansson, 2000; Hoffmann, 2012; Ma, Sandel, & Svenning, 2016; Svenning et al., 2015). Late Quaternary climate change has led to reduced plant species diversity in Arctic islands (Hoffmann, 2012) and some tree extinctions in Europe (Svenning, 2003). Furthermore, late Quaternary climate change has played a major role in shaping plant community compositions (Cabral, Weigelt, Kissling, & Kreft, 2014; Feng et al., 2014; Ma et al., 2016).

Most studies of historical climate changes have focused on macroorganisms (Ma et al., 2016; Normand et al., 2011; Svenning et al., 2015), while the effect of historical climate changes on microorganisms has received little attention (Capo et al., 2016; Delgado‐Baquerizo, Bissett, et al., 2017). Previous studies found that a direct effect of paleoclimate on microbial communities might have occurred in the past (e.g., in response to a severe climatic event), but if the microbial communities response to past climatic change slowly which lead to the consequences of this compositional shift might still be detectable today (Capo et al., 2016; Delgado‐Baquerizo, Bissett, et al., 2017). For example, Delgado‐Baquerizo, Bissett, et al. (2017) found that paleoclimate explained a unique proportion of the variation in soil bacterial richness and community composition across a global scale.

Fungi are a crucial component of soil microorganism communities and exhibit a wide range of lifestyles including mycorrhizal, endophytic, pathogenic, and saprotrophic (Peay, Kennedy, & Bruns, 2008). They play important roles in biodiversity maintenance, community assembly, and ecosystem functioning (Bardgett & van der Putten, 2014; van der Heijden, Bardgett, & van Straalen, 2008). Previous studies have shown that the diversity and composition of soil fungal communities are related to contemporary environmental factors, such as climate, soil, geography, and plant community composition (Gao et al., 2017; Glassman, Wang, & Bruns, 2017; Prober et al., 2015; Talbot et al., 2014; Tedersoo et al., 2014). For example, temperature and precipitation can influence soil fungal communities by affecting their metabolic and growth rates (Bååth, 2001; Hawkes et al., 2011; Malcolm, López‐Gutiérrez, Koide, & Eissenstat, 2008; Pietikäinen, Pettersson, & Bååth, 2005). Plants can influence fungal communities through specific host relationships (Bahram, Harend, & Tedersoo, 2014; Bennett & Cahill, 2016; Nguyen, Williams, et al., 2016; Põlme et al., 2017; Wardle, 2006), and fungal communities can be influenced by soil through the physiological processes of soil biota and nutrient availability (Mexal & Reid, 1973; Peay, Kennedy, & Talbot, 2016; Waldrop, Zak, Blackwood, Curtis, & Tilman, 2006). However, there are few studies investigating the effect of historical climatic change on soil fungal communities (Capo et al., 2016). If historical climate change can be used to explain the current distribution of soil fungal communities, then it may improve our capacity to predict how soil fungal communities will respond to forecasted climate changes in the future.

The late Quaternary climate oscillations in some areas were mild, promoting assemblages with certain community compositions, while others are unstable, tending to select for a different set of communities (Svenning et al., 2015). In general, the temperate region encountered repeated glaciations and a generally cool climate during the Quaternary period, while tropic–subtropical regions were less influenced by glacial cooling, resulting in stronger late Quaternary climate oscillations in temperate regions than in tropic–subtropical areas (Feng, Mao, Sandel, Swenson, & Svenning, 2016; Feng et al., 2014; Loarie et al., 2009; Ruddiman, 2014). Given the stronger effect of late Quaternary climate change on plant communities in temperate regions than in tropic–subtropical areas (Feng et al., 2016, 2014), the late Quaternary climate change may similarly have a stronger effect on the soil fungal community (either directly or indirectly via impacts on plant communities) in temperate than in tropic–subtropical regions. In addition, contemporary environmental variables including climate, plant community structure, and soil physicochemical properties vary from temperate to tropic regions, and these changes result in different fungal biogeographical patterns across ecosystems (Andrew et al., 2018; Bahram et al., 2013; Tedersoo et al., 2012). For example, saprotrophic and ectomycorrhizal (EM) fungal communities have shown contrasting biogeographical patterns driven by different environmental variables from temperate to tropic forests (Andrew et al., 2018; Bahram et al., 2013; Tedersoo et al., 2012).

Forest ecosystems are widely distributed across temperate, subtropical, and tropic regions in China, and they differ widely in plant community composition and diversity. They also encompass a wide range of soil types and climate zones (López‐Pujol & Ren, 2010). Furthermore, with increasing latitude, the magnitude of late Quaternary climate oscillations increases (Feng et al., 2016, 2014). In this study, we investigated soil fungal communities in Chinese temperate, subtropical, and tropic forests from the north to the south (geographic distance up to ~5,500 km) using Illumina MiSeq sequencing to test the following questions (a) late Quaternary climate change affects fungal community composition and fungal richness, (b) late Quaternary climate change influences the fungal community composition of temperate forests more than that of tropic–subtropical forests, and (c) the main contemporary environmental factors affecting fungal community in the different forests are different.

## 2 MATERIALS AND METHODS

### 2.1 Study site and sampling

**Figure 1:**
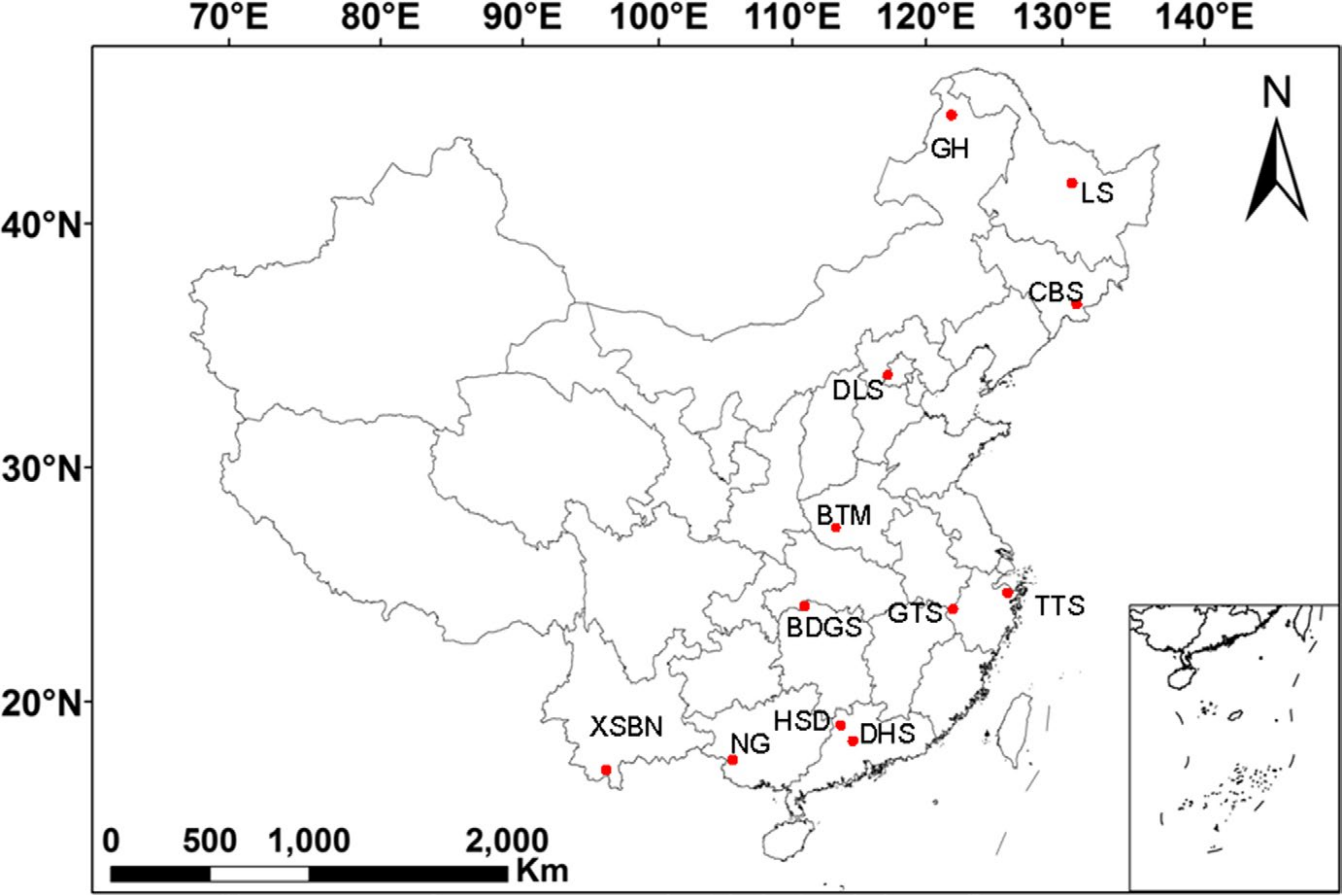
Geographic distribution of sampling sites in China. Temperate forests include Genhe (GH), Liangshui (LS), Changbaishan (CBS), Donglingshan (DLS), and Baotianman (BTM); subtropical forests include Gutianshan (GTS), Badagongshan (BDGS), Tiantongshan (TTS), Heishiding (HSD), and Dinghushan (DHS); tropic forests include Nonggang (NG) and Xishuangbanna (XSBN)

This study was conducted in 12 sites, including temperate (5 sites), subtropical (5 sites), and tropic (2 sites) forests in China (Figure 1). Longitude, latitude, and altitude at the sites were provided by the Chinese Forest Biodiversity Monitoring Network (CForBio), and estimates of the mean annual temperature (MAT) and mean annual precipitation (MAP) were obtained from the WorldClim global climate data (www.worldclim.org) set with a resolution of 2.5 min. In each site, one permanent forest plot (varying from 15–25 ha) was constructed by the CForBio. In each plot, all trees with a diameter ≥1 cm at breast height have been mapped, identified, and measured. During June to October in 2014, 20 quadrats (20 × 20 m) in each plot were randomly selected, and ten soil cores (10 cm in depth, 5 cm in diameter) were randomly collected and mixed as one composite sample in each quadrat. A total of 240 soil samples were therefore used in this study. Soil samples were immediately packed in an ice box and transported to the laboratory. Fresh soil samples were sieved through a 2‐mm sieve to remove roots and debris. Subsamples for DNA extraction were stored at −80°C until analysis, and the remaining subsamples were air‐dried for soil analysis. The information of geographic and current climatic variables of each study site is summarized in Table S1.

### 2.2 Soil properties

Soil properties were measured as follows: pH was measured with a glass electrode at a 1:2.5 (w/v) soil‐to‐water ratio; total carbon (C) and total nitrogen (N) with an Elementar Vario EL III (Elementar Analysensysteme GmbH, Germany); total phosphorus (P), total calcium (Ca), and total magnesium (Mg) with an Inductively Coupled Plasma‐Atomic Emission Spectrometer (ICP‐AES) (iCAP 6300, Thermo Jarrell Ash Co.); particle size distribution (PSD) with an X‐ray diffractometer (Rigaku D/Max 2550pc, Rigaku Corporation) at a voltage of 40 kV and current of 300 mA.

### 2.3 Molecular analysis

Genomic DNA was extracted from 0.25 g frozen soil using the PowerSoil DNA isolation kit (MoBio Laboratories, Inc.) according to the manufacturer's instructions. DNA concentration of each sample was measured using a NanoDrop ND‐1000 Spectrophotometer (NanoDrop Technologies). The fungal internal transcribed spacer 1 (ITS1) region was amplified using a two‐step PCR procedure for Illumina MiSeq sequencing. The first amplification of the entire ITS region with primers ITS1F (White, Bruns, Lee, & Taylor, 1990) and ITS4 (Gardes & Bruns, 1993) was carried out in a final 25 μl reaction solution including 2.5 μl 10 × buffer, 1.5 mM MgSO_4_, 200 μM of each dNTP, 0.75 μM of each primer, 0.5 U KOD‐plus‐Neo polymerase (Toyobo), and *ca*. 10 ng of template DNA. The thermal cycling consisted of an initial denaturation at 95°C for 5 min, followed by 30 cycles of denaturation at 94°C for 50 s, annealing at 52°C for 1 min, and extension at 68°C for 1 min, followed by a final extension at 68°C for 10 min. The product of the first amplification was diluted (×40) with sterilized deionized water and used as the template for the second PCR using the ITS5 and ITS2 primers (White et al., 1990) linked to a barcode sequence (6 base pairs (bp) in length). Conditions for the nested PCR were the same as the first‐round PCR. PCR products were purified using a gel purification kit (Axygen), and 50 ng of DNA from each of the 240 samples was pooled and adjusted to 10 ng/µl. A sequencing library was constructed by adding Illumina sequencing adaptor (5′‐GATCGGAAGAGCACACGTCTGAACTCCAGTCACATCACGATCTCGTATGCCGTCTTCTGCTTG‐3′) to the PCR products using an Illumina TruSeq DNA PCR‐Free Library Preparation Kit (Illumina) following the manufacturer's instructions. The library was then sequenced on an Illumina MiSeq PE250 instrument (running the 2 × 250 bp chemistry) in the Chengdu Institute of Biology, Chinese Academy of Sciences, China.

### 2.4 Sequence processing

Clean ITS1 sequences were obtained from raw sequences after quality control filtering using QIIME v. 1.7.0 (Caporaso et al., 2010). Quality control included removing low‐quality reads with no valid primer or barcode sequence, containing ambiguous bases, or an average quality score <20. The ITS1 region of each remaining sequence was extracted using the fungal ITSX software package (Bengtsson‐Palme et al., 2013), and potential chimeras were subsequently detected using the chimera.uchime command in Mothur version 1.31.2 (Schloss et al., 2009) by comparison with entries in the unified system for the DNA‐based fungal species linked to the classification (UNITE) database (Kõljalg et al., 2013). Nonchimeric ITS1 sequences were clustered into different operational taxonomic units (OTUs) at a 97% sequence similarity level based on the UPARSE pipeline using the USEARCH version 8.0 after discarding replicated sequences and singletons (Edgar, 2013). A representative sequence (the most abundant) of each OTU was selected and searched against the international nucleotide sequence databases collaboration (INSDC) and UNITE databases, using a basic local alignment search tool (BLAST). Fungal OTUs were identified following Tedersoo et al. (2014). Briefly, <e^−50^ values of BLASTn search results were deemed sufficiently reliable for robustly assigning sequences to the fungal kingdom, sequences yielding values between e^−20^ and e^−50^ were manually checked against the 10 best matches for accurate assignment, and those with >e^−20^ values were excluded. Sequence identities of 90%, 85%, 80%, and 75% were used as criteria for assigning OTUs to genera, families, orders, and classes, respectively. All Glomeromycota were considered to be arbuscular mycorrhizal (AM) fungi, and taxa were considered to be EM fungi if they best matched any sequences of known EM lineages (Tedersoo & Smith, 2013). If different functional categories were present within a specific genus, we chose the dominant group (i.e., if >75% of species were assigned to a specific category) or considered its ecology unknown (if ˂75% of species were assignable to a single category). Fungal OTUs were assigned to six functional groups; AM, EM, saprotrophic, pathogenic, animal parasitic, and mycoparasitic following Tedersoo et al. (2014) and the most updated list in FunGuild (Nguyen, Song, et al., 2016; Table S2). The number of sequences per sample was normalized to the smallest sample size using the sub.sample command in Mothur to eliminate the effect of different read numbers. Representative fungal OTU sequences have been submitted to the European Nucleotide Archive (ENA) under Accession nos. LT986405–LT998319.

### 2.5 Late Quaternary climate change data

Among the late Quaternary climate change variables, the velocity of MAT and MAP is a measure of the local rate of displacement of climatic conditions over Earth's surface (Loarie et al., 2009; Sandel et al., 2011); the anomaly of MAT and MAP represents temporal difference in extreme events such as extreme high or low in temperature and precipitation (Garcia, Cabeza, Rahbek, & Araújo, 2014). The velocity and anomaly of MAP and MAT metrics have been widely applied to examine the effect of late Quaternary climate change on macroorganisms (Feng et al., 2014; Garcia et al., 2014; Sandel et al., 2011). Therefore, we used the velocity and anomaly of MAT and MAP as representatives of the late Quaternary climate change variables. We computed the velocity and anomaly for MAT and MAP between the last glacial maximum (LGM; ~21,000 years ago) and the present (1950–2000), with a resolution of 2.5 arc minutes (Sandel et al., 2011). Velocity and anomaly calculations require estimates of climate (at least two time points). To summarize LGM climate, we used estimates of past MAT and MAP from the Paleoclimate Modelling Intercomparison Project Phase II (Braconnot et al., 2007), using the means of the CCSM3 (Collins et al., 2006) and MIROC3.2 (K‐1 model developers, 2004) simulations. The information of paleoclimate of each study site is summarized in Table S1.

### 2.6 Statistical analyses

We analyzed the effect of contemporary (plant, soil, current climate, and geographic distance) and paleoclimatic environmental factors on the richness and composition of total soil fungi and the various functional groups in temperate, tropic–subtropical, and all forest ecosystems. All statistical analyses were carried out in R version 3.3.1 (R Development Core Team, 2017). The *p*‐values were corrected using the Bonferroni method (*p*_adj_). To reduce the ten measured soil variables to a convenient number, principal component (PC) analysis was applied, using the rda function in the VEGAN package (Oksanen et al., 2007). The first four soil PCs (largely reflecting variables of each PC in parentheses) explained 86.8% of the total variation of soil variables: PC1 (total P, C: P ratio, N: P ratio, total Ca, total Mg, and pH) 47.5%, PC2 (total C and C: N ratio) 19.7%, PC3 (total N and PSD) 10.3%, and PC4 (N: P ratio) 9.3% (Table S3). The spatial principal coordinate of neighbor matrices (PCNM) vectors with positive eigenvalues were obtained based on the transformation of geographic distance (latitude and longitude) using the PCNM command in the PCNM package (Dray, Legendre, & Peres‐Neto, 2006). Rarefaction curves of the observed fungal OTUs were calculated in each plot (including 20 quadrats), using the specaccum function in the VEGAN package (Oksanen et al., 2007). In order to estimate the importance of paleoclimatic (MAT velocity, MAP velocity, MAT anomaly, and MAP anomaly) and contemporary environmental (species richness and basal area of total, EM and non‐EM plants, soil PC1–PC4, MAP, MAT, spatial PCNM vectors, and altitude) variables to fungal richness, we conducted random forest models analysis using the randomforest package (Liaw & Wiener, 2002). We also assessed the significance of both the model and each predictor with the rfutilities (Evans & Murphy, 2016) and rfpermute (Archer, 2016) packages, respectively. To quantify the effects of paleoclimatic, contemporary environmental variables, and their interactions on richness of total soil fungi and the various functional groups, we employed linear mixed‐effects models to control for the random effect of sites, using the LME4 package (De Boeck et al., 2011). Given the large number of potential predictor variables relative to the number of sites, we employed forward stepwise model selection to obtain a reduced model.

Pairwise modified Raup‐Crick dissimilarity matrices were calculated to depict the community compositions that were independent of the variation in richness of fungi and plants (Chase, Kraft, Smith, Vellend, & Inouye, 2011). Pairwise Euclidean dissimilarity matrices were calculated for the plant (species richness and basal area of total, EM and non‐EM plants), spatial (altitude and geographic distance), soil (PC1‒PC4), current climatic (MAP and MAT), and paleoclimatic (MAT velocity, MAP velocity, MAT anomaly, and MAP anomaly) variables. The fungal and plant dissimilarity matrices were subjected to principal coordinate (PCo) analysis using the cmdscale command in the VEGAN package (Oksanen et al., 2007). We selected the PCo vectors that could cumulatively explain 100% of the variation of the fungal and plant dissimilarity matrices for variation partitioning (Gower, 1966; Legendre & Anderson, 1999). The variations of total composition of soil fungi and the various functional groups (PCo vectors) were partitioned among the plant, soil, current climatic, paleoclimatic, and spatial (altitude and PCNM vectors) variables using the varpart command in the VEGAN package (Oksanen et al., 2007). The variables within each category were forward‐selected using the forward.sel command in the PACKFOR package (Dray, Legendre, & Blanchet, 2009), then subjected to variation partitioning (Legendre & Legendre, 2012). In addition, permutational multivariate analysis of variance (PerMANOVA) using the adonis command in the VEGAN package (Oksanen et al., 2007) was performed to disentangle the relative effect of paleoclimate, plant, current climate, soil, space (altitude and PCNM vectors), and their interactions between these variables on total soil fungi and the various functional groups.

Furthermore, we constructed structural equation models (SEMs) to disentangle the direct and indirect effects of paleoclimate, plant, current climate, soil, and space on richness and composition of the total soil fungi and the various functional groups. For the fungal richness, we constructed the SEMs using multilevel SEM (Shipley, 2009) implemented in the PIECEWISESEM package (Lefcheck, 2016). This approach integrates a set of linear mixed‐effects models to test the direct and indirect effects. Basis set construction, goodness‐of‐fit tests and parameter estimation were conducted using functions from the PIECEWISESEM package. For the fungal community composition, we constructed the SEMs using Shipley's d‐separation method of generalized causal path analysis (Shipley, 2009) based on the multiple regression on distance matrices (MRM) according to Barnes et al. (2016). All the paleoclimatic and contemporary environmental matrices were subjected to MRM, with forward selection until *p*_adj_ < 0.05 for all variables using the MRM function in the ECODIST package (Goslee & Urban, 2007). In order to construct the path model, we identified the basis set *B_U_* of independence claims that were implied by a hypothetical causal model. The independence claims in *B_U_* describe the *p_i_* probability that variable pairs (*X_i_*, *X_j_*) are independent conditional on the variable set *Z*, which is a direct cause of either *X_i_* or *X_j_*. The combined *p_i_* of the full model was calculated as follows:

$$C=-2\sum_{i=1}^{k}\ln(p_{i})$$

where *C* value was then compared with a chi‐square (*χ* ^2^) distribution with 2*k* degrees of freedom (Shipley, 2009). The resulting probability, *p*, indicates whether the data depart significantly from what would be expected under such a causal model. A model is rejected if the resulting *p* is smaller than the specified α‐level (in this case, *α* = 0.05). As such, if *p *> 0.05, the causal model is considered to provide a good fit to the data (Shipley, 2009). All MRM models were performed with 10,000 permutations to ensure stable estimations of the *p*‐values that were used to calculate the *C* statistic in the path models. To assess the relative importance of paleoclimatic and contemporary environmental variables for driving the composition of total soil fungal communities and the various functional groups, we calculated range‐standardized coefficients for each predictor variable (Grace, 2006). Specifically, this is a standardization of raw coefficients *β_xy_* expressing the effect of x on y, whereby the range‐standardized coefficient *β*std*_xy_* = *β_xy_* × (*x*_max_ − *x*_min_)/(*y*_max_ − *y*_min_), where the maximum and minimum values are the largest and smallest calculated dissimilarity values from the distance matrices. This method of coefficient standardization yields dimensionless coefficients that can be interpreted as the proportional change in *y* across the range of *x* after controlling for all other predictors in the model.

## 3 RESULTS

### 3.1 General characterization of Illumina sequencing data

After removing 280,429 sequences that did not meet the quality criteria, the remaining nonchimeric ITS1 sequences (8,427,416 in total) were clustered into 25,757 nonsingleton OTUs at a 97% sequence similarity level. Of these 25,757 OTUs, 18,171 (representing 8,078,161 reads) were identified as fungal. As the fungal read numbers ranged from 5,290 to 88,715 in the 240 samples, the dataset was normalized to 5,290, resulting in a normalized dataset containing 14,911 fungal OTUs (1,267,912 reads; Table S2). The represented fungi included 8,640 Ascomycota, 4,791 Basidiomycota, 200 Cryptomycota, 647 Zygomycota, 123 Glomeromycota, 117 Chytridiomycota, and 393 unknown fungi (Figure S1a). Of the 14,911 fungal OTUs, 10,021 were assigned to six fungal functional groups, that is, saprotrophic fungi (6,149), EM fungi (2,709), pathogenic fungi (724), animal parasitic fungi (194), AM fungi (123), and mycoparasitic fungi (122) (Figure S1b). The abundant fungal functional groups, such as saprotrophic, EM, and pathogenic fungi, were included in further analysis. Rarefaction curves of the observed fungal OTU richness did not reach an asymptote in any site (Figure S2).

### 3.2 Fungal richness in temperate, tropic–subtropical, and all forests

The OTU richness of total, saprotrophic, EM, and pathogenic fungi was 3,812 ± 171, 1,624 ± 111, 919 ± 50.8, and 170 ± 13.7 (site average ± standard deviation) in temperate forest and 4,214 ± 595, 1,798 ± 111, 740 ± 85.3, and 229 ± 55.1 in tropic–subtropical forest. The random forest models for samples across all forest types showed that the most important variable for total fungal richness, as well as richness of saprotrophic and pathogenic fungi, was soil PC1 and followed by total plant species richness, while the most important variable of EM fungal richness was EM plant species richness (Table 1). For samples from tropic–subtropical forest, the most important variable of total, saprotrophic, and pathogenic fungal richness was soil PC1 and followed by total plant species richness, but for samples from temperate forest the most important variables of total, saprotrophic, and pathogenic fungal richness were soil PC1 and/ or soil PC2 in tropic–subtropical forest (Table 1). However, the most important variable of EM fungal richness was EM plant species richness in temperate and tropic–subtropical forests (Table 1). The linear mixed‐effects models showed that total fungal richness was mainly determined by total plant species richness and soil PC1, and the same is true for saprotrophic and pathogenic fungal richness, while EM fungal richness was largely determined by EM plant species richness across all forests (Table 2). Furthermore, total and saprotrophic fungal richness were significantly related to soil PC1 in temperate forest, but to soil PC1 and interaction between total plant species richness and soil PC1 in tropic–subtropical forest (Table 2). In addition to soil PC1, pathogenic fungal richness was significantly related to soil PC2 in tropic–subtropical forest, but not in temperate forest (Table 2). However, EM fungal richness was significantly related to EM plant species richness in temperate and tropic–subtropical forests (Table 2).

**Table 1:** Results of total, saprotrophic, pathogenic, and ectomycorrhizal (EM) fungal richness predicted by significantly environmental variables in temperate, tropic–subtropical, and all forests as explored by random forest models

Fungal richness	Variable	Importance	*p* value	Model *R* ^2^	Model *p* value
Temperate forest					
Total fungi	Soil PC1	16.832	0.006	0.042	0.001
Saprotrophic fungi	Soil PC1	27.17	0.014	0.31	0.001
Pathogenic fungi	Soil PC1	3.318	0.034	0.032	0.001
EM fungi	EM plant species richness	17.09	0.01	0.065	0.001
Tropic–subtropical forest					
Total fungi	Soil PC1	18.75	0.002	0.068	0.001
	Total plant species richness	18.82	0.008		
Saprotrophic fungi	Soil PC1	22.435	0.002	0.127	0.001
	Total plant species richness	20.4	0.006		
Pathogenic fungi	Soil PC1	39.381	0.002	0.311	0.001
	Soil PC2	16.866	0.04		
EM fungi	EM species richness	28.455	0.004	0.034	0.001
All forest					
Total fungi	Soil PC1	36.68	0.002	0.1	0.001
	Total plant species richness	38.55	0.004		
Saprotrophic fungi	Soil PC1	44.9	0.002	0.133	0.001
	Total plant species richness	37.757	0.004		
Pathogenic fungi	Soil PC1	40.032	0.002	0.333	0.001
	Total plant species richness	53.437	0.006		
EM fungi	EM plant species richness	44.424	0.008	0.214	0.001

Note

Soil PC1, total phosphorus (P), total carbon (C): P ratio, nitrogen (N): P ratio, total calcium (Ca), total magnesium (Mg), and pH; soil PC2, total C, and C: N ratio.

**Table 2:** Results of total, saprotrophic, pathogenic, and ectomycorrhizal (EM) fungal richness predicted by plant and abiotic variables in temperate, tropic–subtropical, and all forests as explored by linear mixed‐effects models controlling random effects of sites

Fungal richness	Independent variable	Slope	*SE*	*df*	*t*	*p*_adj_
Temperate forest						
Total fungi	Soil PC1	159.277	48.057	93	3.314	0.005
Saprotrophic fungi	Soil PC1	96.126	27.901	93	3.445	0.004
Pathogenic fungi	Soil PC1	12.198	4.326	93	2.820	0.024
EM fungi	EM plant species richness	6.164	2.887	93	2.135	0.035
Tropic–subtropical forest						
Total fungi	Soil PC1	658.048	173.701	131	3.788	0.001
	Total plant species richness × soil PC1	−12.868	4.736	131	−2.717	0.030
Saprotrophic fungi	Soil PC1	313.816	85.456	131	3.672	0.001
	Total plant species richness × soil PC1	−5.965	2.319	131	−2.573	0.045
Pathogenic fungi	Soil PC1	30.548	3.802	131	8.036	0.000
	Soil PC2	−12.623	3.289	131	−3.838	0.001
EM fungi	EM plant species richness	4.854	1.721	132	2.820	0.022
All forest						
Total fungi	Total plant species richness	4.164	0.960	226	4.336	0.000
	Soil PC1	168.424	35.746	226	4.712	0.000
Saprotrophic fungi	Total plant species richness	1.808	0.494	226	3.662	0.001
	Soil PC1	90.207	19.098	226	4.723	0.000
Pathogenic fungi	Total plant species richness	0.373	0.103	226	3.608	0.002
	Soil PC1	15.545	4.444	226	3.498	0.002
EM fungi	EM plant species richness	4.933	1.680	227	2.937	0.015

Note

Soil PC1, total phosphorus (P), total carbon (C): P ratio, total calcium (Ca), total magnesium (Mg), and pH; soil PC2, total C and C: total nitrogen (N) ratio.

**Figure 2:**
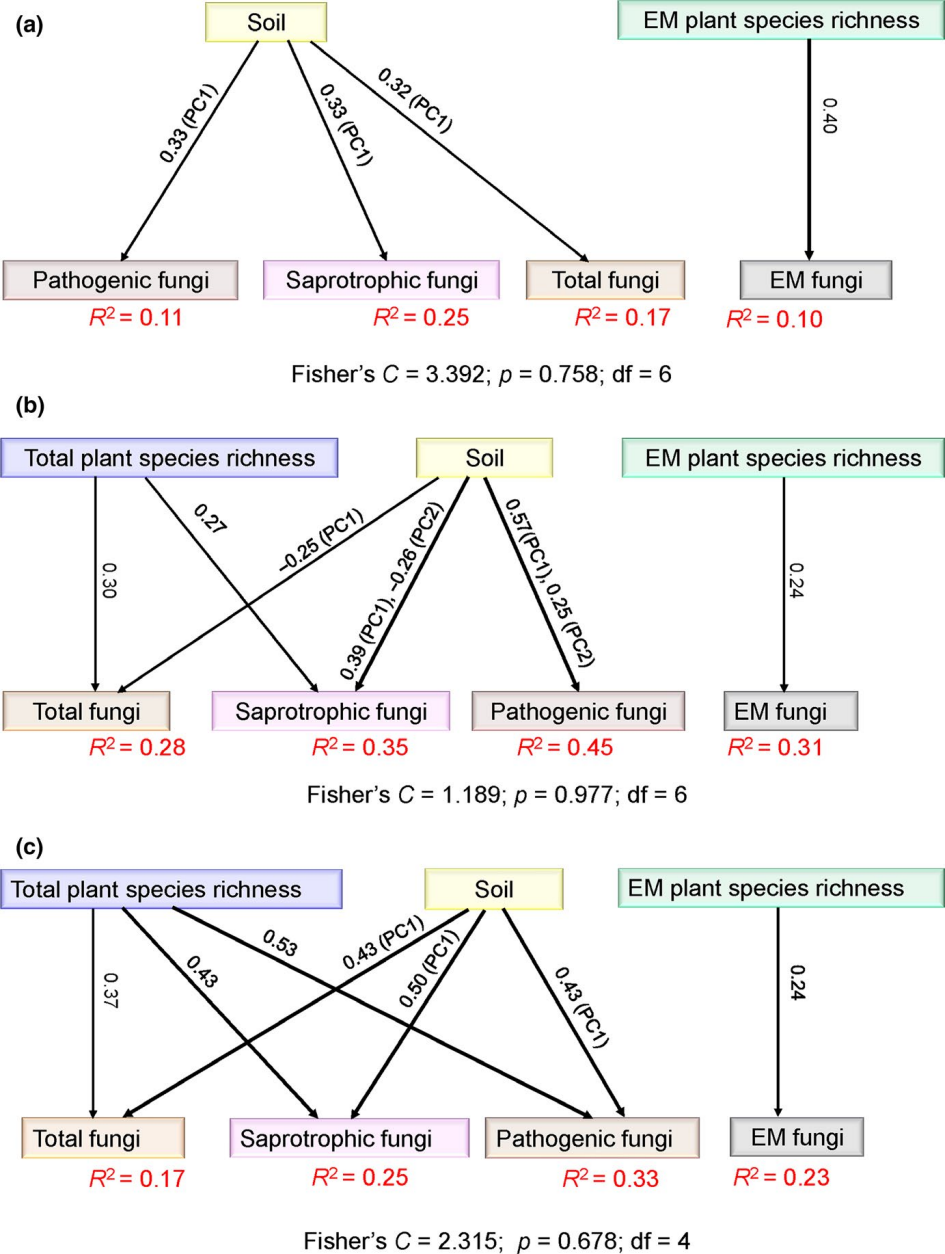
Structural equation models (SEMs) accounting for the direct and indirect effects of contemporary environment on fungal richness. (a)–(c) are the SEMs results showing the direct and indirect effects of contemporary environment on total, saprotrophic, pathogenic, and ectomycorrhizal (EM) fungal richness in temperate, tropic–subtropical, and all forests, respectively. The width of the lines represents the strength of the relationship (*p* < 0.05). Numbers above the lines indicate path coefficients. *R* ^2^ values represent the proportion of variance explained. Soil PC1, total phosphorus (P), total carbon (C): P ratio, total calcium (Ca), total magnesium (Mg), and pH. Soil PC2, total C, and C: total nitrogen (N) ratio

The final SEMs showed that total plant species richness had a direct effect on total, saprotrophic, and pathogenic fungal richness in all forests, and on total and saprotrophic fungal richness in tropic–subtropical forest (Figure 2). EM plant species richness had a direct effect on EM fungal richness in temperate, tropic–subtropical, and all forests (Figure 2). Soil PC1 had a direct effect on total, saprotrophic, and pathogenic fungal richness in temperate, tropic–subtropical, and all forests; in contrast, soil PC2 had a direct effect on saprotrophic and pathogenic fungal richness in tropic–subtropical forest (Figure 2). Taking these findings from the linear mixed‐effects models and SEMs analyses as a whole, none of the variables describing late Quaternary climate change had significant effects on total and functional group fungal richness in temperate, tropic–subtropical, and all forests.

### 3.3 Fungal community composition in temperate, tropic–subtropical, and all forests

**Figure 3:**
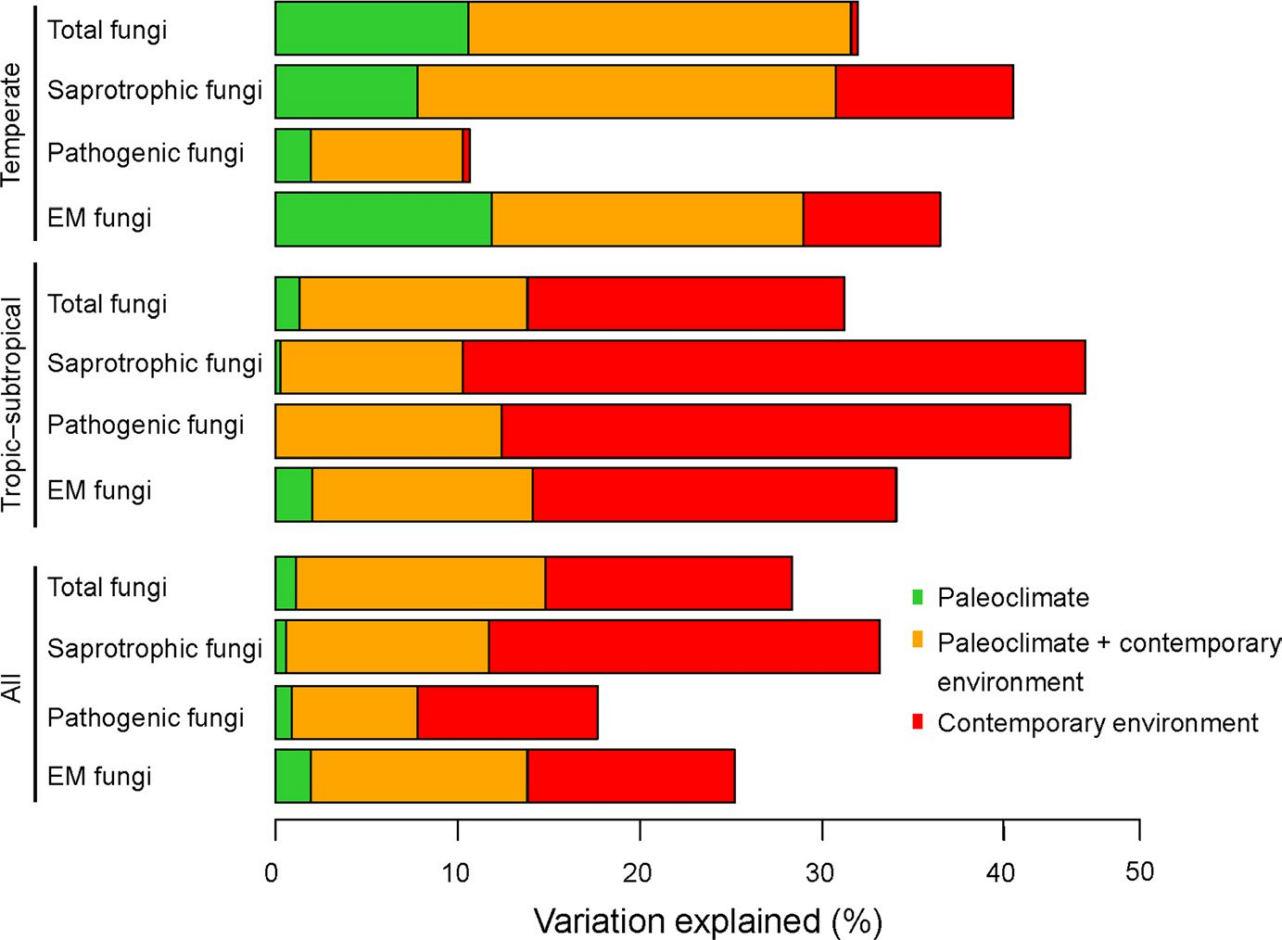
Relative contribution of the different predictors used to model total and functional group fungal community compositions in temperate, tropic–subtropical, and all forests. Panels represent results from variation partitioning modelling aiming to identify the percentage variance of total, saprotrophic, pathogenic, and ectomycorrhizal (EM) fungal community compositions explained by paleoclimatic and contemporary environmental (current climate, space, plant, and soil) variables in the temperate, tropic–subtropical, and all forests. Unique and shared variance from the current climate, spatial, plant, and soil variables in predicting total, saprotrophic, pathogenic, and EM fungal community compositions were merged in this figure for simplicity. Note that the variation explained by “paleoclimate + contemporary environment” is additional to the one explained either by paleoclimate only or contemporary environment only. An alternative version of this figure showing the unique and shared variance of each group of predictors can be found in Figure S3

The variation partitioning showed that 29.1%–31.9%, 33.1%–44.3%, 10.0%–42.1%, and 25.1%–35.5% of the variance of total, saprotrophic, pathogenic, and EM fungal community compositions were explained by paleoclimatic and contemporary environmental variables in temperate, tropic–subtropical, and all forests (Figure 3; Figure S3). Furthermore, paleoclimate explained higher variance in community compositions of total (31.6% vs. 13.8%), saprotrophic (30.8% vs. 10.3%), pathogenic (10.3% vs. 10.1%), and EM (29.0% vs. 14.1%) fungi in temperate forest than in tropic–subtropical forest (Figure 3; Figure S3). Some contemporary environmental variables explaining total and functional group fungal community compositions were different in temperate and tropic–subtropical forests (Figure S3). For example, in addition to soil PC1, geographic distance, current climate and total plant community composition, total, and saprotrophic fungal community compositions were explained by soil PC2 in temperate forest but by soil PC3 and PC4 in tropic–subtropical forest (Figure S3). Besides soil PC1, geographic distance, and current climate, pathogenic fungal community composition was related to total plant community composition, soil PC3, and PC4 in tropic–subtropical forest but not in temperate forest (Figure S3). In addition to EM plant community composition, current climate, and geographic distance, EM fungal community composition was related to non‐EM plant community composition, soil PC1, and PC2 in tropic–subtropical forest but not in temperate forest (Figure S3).

PerMANOVA for samples from temperate, tropic–subtropical, and all forests types revealed that 89.6%–94.6%, 89.4%–94.2%, 42.6%–87.4%, and 57.2%–78.6% of the variance of total, saprotrophic, pathogenic, and EM fungal community compositions were explained by paleoclimatic and contemporary environmental variables (Table S4). Furthermore, paleoclimate explained higher variance in community compositions of total (89.6% vs. 0.4%), saprotrophic (89.5% vs. 2.6%), pathogenic (42.6% vs. 3.2%), and EM (48.6% vs. 4.6%) fungi in temperate forest than in tropic–subtropical forest (Table S4). Some contemporary environmental variables explaining total and functional group fungal community compositions were different in temperate and tropic–subtropical forests (Table S4). For example, in addition to total plant community composition, saprotrophic fungal community composition was explained by soil PC2 in temperate forest but not in tropic–subtropical forest (Table S4). Pathogenic fungal community composition was explained by total plant community composition, PCNM (PCNM1‐3), and soil (PC1‐3) in tropic–subtropical forest but not in temperate forest (Table S4). In addition to EM plant community composition, EM fungal community composition was explained by non‐EM plant community composition and PCNM (PCNM1‐3) in tropic–subtropical forest, but not in temperate forest (Table S4).

**Figure 4:**
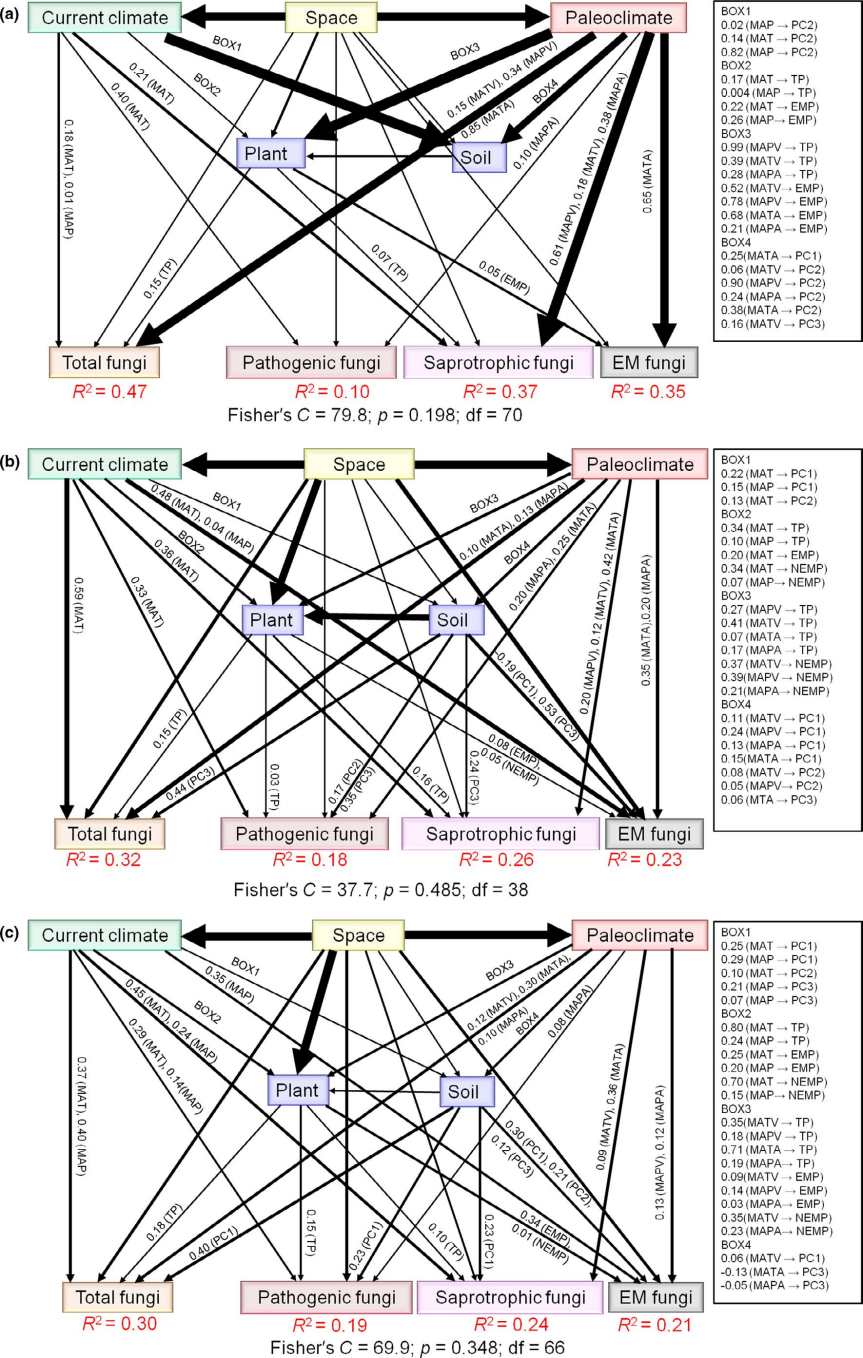
Structural equation models (SEMs) accounting for the direct and indirect effects of paleoclimate and contemporary environment on fungal community composition. (a)–(c) are the SEMs results showing the direct and indirect effects of paleoclimate and contemporary environment (current climate, space, plant, and soil) on total, saprotrophic, pathogenic, and ectomycorrhizal (EM) fungal community compositions in temperate, tropic–subtropical, and all forests, respectively. The width of the lines represents the strength of the relationship (*p* < 0.05). Numbers above and below the lines indicate path coefficients, and only the path coefficients between current climate, paleoclimate, plant, soil, and fungal community composition were shown in this figure. *R* ^2^ values represent the proportion of variance explained. We grouped the different categories of predictors (soil, paleoclimate, current climate, and space) in the same box for graphical simplicity. Other details can be found in the Table S5

The final SEMs for samples from temperate, tropic–subtropical, and all forests types demonstrated that paleoclimate, current climate, and space had direct effects on total, saprotrophic, pathogenic, and EM fungal community compositions (Figure 4; Table S5). Furthermore, for samples from temperate and tropic–subtropical forests paleoclimate, current climate and space had indirect effects on total and saprotrophic fungal community compositions through total plant community composition and soil (Figure 4; Table S5). The same trend was observed when all forest types were analyzed together (Figure 4; Table S5). EM fungal community composition was affected indirectly by paleoclimate, current climate, and space through EM and non‐EM plant community compositions and soil in tropic–subtropical and all forests, but through EM plant community composition and soil (via EM plant community composition) in temperate forest (Figure 4; Table S5). Pathogenic fungal community composition was affected indirectly by paleoclimate, current climate, and space through total plant community composition and soil in tropic–subtropical and all forests, but not in temperate forest (Figure 4; Table S5).

## 4 DISCUSSION

This study has shown that the richness of total soil fungi and various functional groups were not affected by late Quaternary climate change. While there is a paucity of studies to compare this to, it has been shown that fungal richness in the sediment of two European lakes was not related to the past two millennia of paleoclimatic variations (Capo et al., 2016). Some fungal taxa increased in richness but some decreased in richness under late Quaternary climate change which might result in the overall fungal richness remaining unchanged, as expectation that different fungal taxa have different response to climatic changes (Newsham et al., 2016). This is different from the effect of Quaternary climate change on bacterial species richness in a global scale (Delgado‐Baquerizo, Bissett, et al., 2017). The difference might be explained partly by the different traits between fungi and bacteria. Soil fungi are more resistant to climatic changes than bacteria (Barnard, Osborne, & Firestone, 2013; De Vries et al., 2012; De Vries & Shade, 2013). This trait of fungi may reduce species' vulnerability to range loss, avoid extinction risk, and improve persistent ability of fungi encountering the late Quaternary climate change, resulting that fungal richness was not related to late Quaternary climate change. Alternatively, bacterial research was conducted in a global scale, but this study was carried out in a regional scale, as the response of microbial richness depends on biogeographical scales (Bahram et al., 2016; Martiny, Eisen, Penn, Allison, & Horner‐Devineet, 2011).

We found that total and functional group fungal richness were significantly affected by contemporary environmental factors, such as soil nutrients, pH, and/or plant species richness in temperate, tropic–subtropical, and all forests, as reported in some previous studies (Gao et al., 2017; Glassman et al., 2017; Peay, Baraloto, & Fine, 2013; Prober et al., 2015; Tedersoo et al., 2014). However, total and functional group fungal richness were affected by some different contemporary environmental factors in temperate and tropic–subtropical forests, as reported in Tedersoo et al. (2014). For example, we found that the interaction between total plant species richness and soil (nutrients and pH) affected the total and saprotrophic fungal richness in tropic–subtropical forest. It is highly possible that plants can modify soil environments through litter or root exudates and soil environments can affect plants in ways resulting in plant–soil feedback that can influence total and saprotrophic fungal richness in tropic–subtropical forest (Erlandson, Wei, Savage, Cavender‐Bares, & Peay, 2018). In addition, the soil C effect on pathogenic fungal richness in tropic–subtropical forests may be because soil C was linked to the plant species diversity in tropic forests (Hooper et al., 2000).

Our results showed that total and functional group fungal community compositions were affected by late Quaternary climate change in temperate, tropic–subtropical, and all forests. Similarly, fungal community composition in the sediment of two European lakes was related to the past two millennia of paleoclimatic variations (Capo et al., 2016). The relationship between late Quaternary climate change and soil fungal community composition may be caused by altering the relative abundance of fungi (Capo et al., 2016), as reported in previous studies that regions where the late Quaternary climate oscillations were similar tend to promote assemblages with similar certain community compositions (Svenning et al., 2015). This also suggests that Quaternary climatic oscillations may produce disequilibrium with current climate if species that were favored under historical conditions but not the current conditions are extirpated slowly, or if species favored by the new conditions are slow to colonize (Svenning & Sandel, 2013). Alternatively, the influence of late Quaternary climate change was mediated by effects on the plant community (Feng et al., 2016; Kissling et al., 2012) and soil (Delgado‐Baquerizo, Eldridge, et al., 2017), which in turn drive changes in fungal community composition (Nguyen, Williams, et al., 2016; Tedersoo et al., 2014), as our SEMs demonstrated (Figure 4).

Furthermore, we found that late Quaternary climate change had a higher effect on the total and functional group fungal community compositions in temperate forest than in tropic–subtropical forest, which is consistent with the effect of late Quaternary climate change on macroorganism community composition (Feng et al., 2014; Svenning et al., 2015). This may be because late Quaternary climate change was stronger in temperate regions than in tropic–subtropical areas (Feng et al., 2016, 2014; Ruddiman, 2014). Across the study sites, paleoclimatic variations (MAT velocity, MAP velocity, MAT anomaly, and MAP anomaly) were 83.6%–148% higher in temperate forest than in tropic–subtropical forest (Table S6), which can directly exert greater effects on soil fungal community composition in temperate forest than in tropic–subtropical forest. Furthermore, the complex topography in tropic–subtropical forests may have buffered this region against historical climate changes (López‐Pujol & Ren, 2010), resulting in a smaller effect of late Quaternary climate change on the fungal community composition. Besides, higher Quaternary climate oscillations in the unstable areas would result in a more phylogenetically clustered plant community (Kissling et al., 2012), which influences soil fungal community composition (Barberán et al., 2015). The importance of this result lies in the fact that late Quaternary climate change can be used to better understand and predict the response of soil fungal community composition to future climate changes.

In addition to late Quaternary climate change, we found that contemporary environmental factors, such as plant community composition, soil, current climate, and space, affected total and functional group fungal community compositions in temperate, tropic–subtropical, and all forests, as reported in previous studies (e.g., Barberán et al., 2015; Gao et al., 2017; Tedersoo et al., 2016; Talbot et al., 2014). However, some different contemporary environmental variables affected total and functional group fungal community compositions in temperate and tropic–subtropical forests. For example, we found that total, saprotrophic, pathogenic, and EM fungal community compositions were related to soil C or no variables in temperate forest, but to soil nutrients, PSD, or pH in tropic–subtropical forest. In the study sites, the range of soil C concentration (3.08–30.39 g/kg vs. 1.4–24.78 g/kg) was wider in temperate forest than in tropic–subtropical forest, but the ranges of soil nutrient concentrations (0.23–33.93 g/kg vs. 0.02–14.53 g/kg), pH (3.31–6.87 vs. 4.4–7.22), and PSD (2.44–2.72 vs. 2.42–2.68) were wider in tropic–subtropical forest than in temperate forest. The wider ranges of soil variables would provide more niche spaces to promote species coexistence of fungal community in different forest ecosystems (Peay et al., 2017). In addition to soil variables, pathogenic fungal community composition was related to plant community composition in tropic–subtropical forests in this study. It is possible that pathogenic fungi as host‐specific enemies would have stronger effect in plant species‐rich tropic–subtropical regions than in plant species‐poor temperate areas (Johnson, Beaulieu, Bever, & Clay, 2012). Furthermore, we found that EM fungal community composition was related to non‐EM plant community composition in tropic–subtropical forest. In the study sites, there are higher non‐EM plant species richness and abundance (on average, 83.7% vs. 66.2% of plant species and 74.3% vs. 48.3% of plant individuals) in tropic–subtropical forest than in temperate forest (Table S6), and these EM plant individuals are surrounded by a large number of the non‐EM plants to form many small tree islands which prevent the spread of EM fungi through underground hyphae in tropic–subtropical forest (Gao et al., 2015; Tedersoo, Sadam, Zambrano, Valencia, & Bahram, 2010).

## 5 CONCLUSIONS

This study is the first to show the effect of late Quaternary climate change on soil fungal community composition. Fungal richness was not affected by the late Quaternary climate change, which is inconsistent with the effect of late Quaternary climate change on bacterial diversity. It might be potentially owing to the different traits, such as resistant ability to climatic changes in bacteria and fungi. Late Quaternary climate change influenced fungal community composition and with a stronger signature in temperate forests than in tropic–subtropical forests, which is similar to the effect of late Quaternary climate change on the macroorganism communities. This finding indicates that late Quaternary climatic change can be used to better understand the maintaining mechanism of current soil fungal community composition. Thus, climatic legacy effect should be considered in predicating the soil fungal community composition when assessing the responses of these communities, and the ecosystem services that they provide, to ongoing climate changes.

## CONFLICT OF INTEREST

None declared.

## AUTHOR CONTRIBUTIONS

N.N.J., C.G., and L.D.G. designed the study, and N.N.J., C.G., Y.Z., L.C., B.W.W., X.C.L., Y.L.W., P.P.L., and X.S. collected the soil samples. N.N.J., B.S., and L.D.G. conducted the statistical analyses and wrote the manuscript.

## Data Availability

The representative fungal OTU sequences have been submitted to the European Nucleotide Archive (ENA) under Accession nos. LT986405–LT998319; PRJEB24315.
