# Supplementary material for: Late Quaternary climate change explains soil fungal community composition rather than fungal richness in forest ecosystems
Source: Ecol Evol. 2019 May 9;9(11):6678–92. doi: 10.1002/ece3.5247 (PMC6580281; doi:10.1002/ece3.5247)
Supplement: Supplementary file 1 [file ECE3-9-6678-s001.docx]

Supplementary Information for

**Late Quaternary climatic change explains s****oil fungal community composition rather than richness in forest ecosystems**

Niu-Niu Ji^1,2^, Cheng Gao^1^, Brody Sandel^3^, Yong Zheng^1^, Liang Chen^1^, Bin-Wei Wu^1,2^, Xing-Chun Li^1^, Yong-Long Wang^1,2^, Peng-Peng Lü^1,2^, Xiang Sun^1^, Liang-Dong Guo^1,2^

^1^State Key Laboratory of Mycology, Institute of Microbiology, Chinese Academy of Sciences, Beijing, China

^2^College of Life Sciences, University of Chinese Academy of Sciences, Beijing, China

^3^Department of Biology, Santa Clara University, Santa Clara CA, USA

**Correspondence**

Liang-Dong Guo, State Key Laboratory of Mycology, Institute of Microbiology, Chinese Academy of Sciences, Beijing, China.

Email: guold@im.ac.cn

This file includes:

Figure S1 to Figure S3

Table S1, Table S3, Table S4 and Table S6

Other Supporting Online Information for this manuscript includes the following:

Table S2 and Table S5 as Excel files

**Figure S1.** **Numbers of operational taxonomic units (OTUs) of fungi belonging to indicated phyla (a) and indicated functional groups (b). Percentage of total numbers of OTUs is given in parentheses.**

**Figure S2.** **Rarefaction of observed fungal operational taxonomic units (OTUs) in each site.** GH, Genhe; LS, Liangshui; CBS, Changbaishan; DLS, Donglingshan; BTM, Baotianman; GTS, Gutianshan; BDGS, Badagongshan; TTS, Tiantongshan; HSD, Heishiding; DHS, Dinghushan; NG, Nonggang; XSBN, Xishuangbanna.

**Figure S3. Variation partitioning analyses showing pure and shared effects of plant and soil, current climate, spatial, and paleoclimatic variables on community compositions of total fungi (a**–**c), saprotrophic fungi (d**–**f), pathogenic fungi (g**–**i) and ectomycorrhizal (EM) fungi (j**–**l) in temperate, tropic-subtropical and all forests.** MAT, mean annual temperature; MAP, mean annual precipitation; soil PC1, total phosphorus (P), total carbon (C) : P ratio, nitrogen (N) : P ratio, total calcium (Ca), total magnesium (Mg), and pH; soil PC2, total C and C : N ratio; soil PC3, total N and particle size distribution (PSD); soil PC4, N : P ratio; PCNM, the axis of principal coordinates analysis of neighbor matrix (PCNM), representing potential spatial structure of samples in the three forests.

| **Table S1.** Geographic and climatic variables of each plot in this study | | | | | | | | | | |  |
| --- | --- | --- | --- | --- | --- | --- | --- | --- | --- | --- | --- |
| Forest type | Plot | Latitude (ºN) | Longitude (ºE) | Altitude  (m) | MAT  (ºC) | MAP (mm) | MAT velocity | MAP velocity | MAT anomaly | MAP anomaly | |
| Temperate forest | Genhe (GH) | 50.80 | 121.52 | 450 | -5.30 | 450.00 | 3.32 | 2.43 | 9.80 | 114.50 | |
| Temperate forest | Liangshui (LS) | 47.18 | 128.89 | 480 | -0.30 | 676.00 | 2.28 | 4.25 | 9.90 | 114.50 | |
| Temperate forest | Changbaishan (CBS) | 42.38 | 128.08 | 802 | 3.60 | 700.00 | 4.35 | 5.24 | 8.35 | 61.50 | |
| Temperate forest | Donglingshan (DLS) | 39.97 | 115.43 | 600 | 4.80 | 550.00 | 0.82 | 0.51 | 5.95 | 143.00 | |
| Temperate forest | Baotianmai (BTM) | 33.49 | 111.94 | 1150 | 15.10 | 885.60 | 0.47 | 0.06 | 4.30 | 113.50 | |
| Subtropical forest | Tiantongshan (TTS) | 29.81 | 121.79 | 447 | 16.20 | 1374.70 | 3.35 | 0.00 | 5.15 | -25.50 | |
| Subtropical forest | Badagongshan (BDGS) | 29.76 | 110.09 | 1400 | 11.50 | 2105.40 | 0.79 | 3.38 | 3.80 | 131.00 | |
| Subtropical forest | Gutianshan (GTS) | 29.25 | 118.12 | 581 | 15.30 | 1963.70 | 0.96 | 0.30 | 4.00 | 102.50 | |
| Subtropical forest | Hheishiding (HSD) | 23.50 | 111.88 | 292 | 19.60 | 1743.80 | 1.00 | 0.33 | 2.65 | -34.50 | |
| Subtropical forest | Dinghushan (DHS) | 23.15 | 112.51 | 400 | 20.90 | 1929.00 | 0.40 | 0.37 | 2.60 | -27.00 | |
| Tropical forest | Nonggang (NG) | 22.43 | 106.95 | 260 | 22.00 | 1300.00 | 1.62 | 1.93 | 2.55 | 149.00 | |
| Tropical forest | Xishuangbanna (XSBN) | 21.36 | 101.34 | 700 | 21.80 | 1493.00 | 0.42 | 0.74 | 3.30 | 27.50 | |
| MAT, mean annual temperature; MAP, mean annual precipitation. | | | | | | | | | | |  |

| **Table S3.** Principal component (PC) analysis of soil variables | | | | |
| --- | --- | --- | --- | --- |
| Soil variable | PC1 | PC2 | PC3 | PC4 |
| Total carbon (C) | 1.324 | 1.623 | -0.019 | 0.557 |
| Total nitrogen (N) | 1.487 | 1.062 | -0.796 | 0.874 |
| Total phosphorus (P) | 1.915 | 0.055 | -0.596 | -0.157 |
| C : N ratio | 0.077 | 1.378 | 1.594 | -0.601 |
| C : P ratio | 1.875 | 0.726 | 0.132 | -0.466 |
| N : P ratio | -1.591 | 0.435 | 0.353 | 1.089 |
| Total calcium (Ca) | 1.823 | -0.215 | 0.594 | 0.213 |
| Total magnesium (Mg) | 1.734 | -0.889 | 0.277 | -0.267 |
| Particle size distribution (PSD) | -0.702 | 0.986 | -0.942 | -1.255 |
| pH | 1.642 | -1.209 | 0.194 | 0.068 |
| Eigenvalue | 4.746 | 1.974 | 1.029 | 0.933 |
| Proportion explained | 0.475 | 0.197 | 0.103 | 0.093 |
| Cumulative proportion | 0.475 | 0.672 | 0.775 | 0.868 |

| **Table S4.** Relative importance of paleoclimate and contemporary environment on total, saprotrophic, pathogenic and ectomycorrhizal (EM) fungal community compositions in temperate, tropic-subtropical and all forests as revealed by permutational multivariate analysis of variance | | | | | | | |
| --- | --- | --- | --- | --- | --- | --- | --- |
| Fungal community composition | Variable | *Df* | *SS* | *MS* | *F-statistic* | R^2^adj | *P*-value |
| **Temperate forest** |  |  |  |  |  |  |  |
| Total fungi | Paleoclimate | 4 | 12.670 | 3.167 | 212.850 | 0.896 | 0.001 |
|  | Plant community composition | 4 | 0.146 | 0.036 | 2.770 | 0.006 | 0.032 |
|  | Soil (PC2) | 1 | 0.084 | 0.084 | 6.378 | 0.010 | 0.011 |
| Saprotrophic fungi | Paleoclimate | 4 | 8.166 | 2.041 | 4126.800 | 0.895 | 0.001 |
|  | Soil (PC2) | 2 | 0.247 | 0.123 | 149.890 | 0.010 | 0.001 |
|  | Plant community composition | 3 | 0.076 | 0.025 | 30.790 | 0.006 | 0.001 |
| Pathogenic fungi | Paleoclimate | 4 | 4.314 | 1.078 | 19.226 | 0.426 | 0.001 |
| EM fungi | Paleoclimate | 2 | 8.860 | 4.430 | 40.882 | 0.486 | 0.001 |
|  | EM plant community composition | 5 | 2.374 | 0.475 | 4.381 | 0.086 | 0.001 |
| **Tropic-subtropical forest** | |  |  |  |  |  |  |
| Total fungi | Plant community composition | 8 | 18.194 | 2.274 | 469.810 | 0.855 | 0.001 |
|  | Soil (PC1‒3) | 3 | 1.174 | 0.391 | 26.798 | 0.037 | 0.001 |
|  | Paleoclimate | 1 | 0.231 | 0.231 | 15.818 | 0.004 | 0.001 |
| Saprotrophic fungi | Plant community composition | 8 | 10.446 | 1.306 | 5056.000 | 0.868 | 0.001 |
|  | Paleoclimate | 2 | 0.437 | 0.219 | 30.174 | 0.026 | 0.001 |
| Pathogenic fungi | Plant community composition | 8 | 14.071 | 0.204 | 11.900 | 0.825 | 0.001 |
|  | PCNM (PCNM1‒3) | 3 | 0.647 | 0.216 | 12.585 | 0.017 | 0.001 |
|  | Paleoclimate | 3 | 0.888 | 0.296 | 17.273 | 0.032 | 0.001 |
|  | Soil (PC1‒3) | 3 | 0.173 | 0.058 | 3.361 | 0.008 | 0.028 |
| EM fungi | Non-EM plant community composition | 8 | 14.559 | 1.820 | 52.525 | 0.588 | 0.001 |
|  | PCNM (PCNM1‒3) | 3 | 1.448 | 0.483 | 13.935 | 0.040 | 0.001 |
|  | EM plant community composition | 6 | 2.664 | 0.444 | 12.816 | 0.072 | 0.001 |
|  | Paleoclimate | 4 | 1.766 | 0.442 | 12.745 | 0.046 | 0.001 |
| **All forest** |  |  |  |  |  |  |  |
| Total fungi | PCNM (PCNM1‒4) | 4 | 32.774 | 8.193 | 5020.800 | 0.849 | 0.001 |
|  | Plant community composition | 14 | 3.922 | 0.654 | 400.600 | 0.086 | 0.001 |
|  | Paleoclimate | 4 | 0.951 | 0.238 | 145.600 | 0.011 | 0.001 |
| Saprotrophic fungi | PCNM (PCNM1‒4) | 4 | 18.506 | 4.626 | 29329.800 | 0.848 | 0.001 |
|  | Plant community composition | 10 | 2.633 | 0.263 | 245.000 | 0.085 | 0.001 |
|  | Paleoclimate | 4 | 0.540 | 0.135 | 856.400 | 0.009 | 0.001 |
| Pathogenic fungi | Plant community composition | 14 | 17.370 | 1.241 | 61.958 | 0.570 | 0.001 |
|  | PCNM (PCNM1‒4) | 4 | 4.991 | 1.248 | 62.303 | 0.156 | 0.001 |
|  | Paleoclimate | 4 | 1.763 | 0.441 | 22.014 | 0.045 | 0.001 |
|  | Current climate | 2 | 0.513 | 0.256 | 12.809 | 0.009 | 0.001 |
|  | Soil (PC1‒2) | 2 | 0.228 | 0.114 | 5.679 | 0.008 | 0.002 |
| EM fungi | EM plant community composition | 11 | 25.901 | 2.355 | 79.245 | 0.514 | 0.001 |
|  | PCNM (PCNM1‒4) | 4 | 11.120 | 2.780 | 93.561 | 0.217 | 0.001 |
|  | Paleoclimate | 4 | 2.787 | 0.697 | 23.451 | 0.042 | 0.001 |
|  | Non-EM plant community composition | 17 | 1.725 | 0.101 | 3.416 | 0.005 | 0.001 |
|  | Current climate | 2 | 0.584 | 0.292 | 9.826 | 0.004 | 0.001 |
|  | Soil (PC1‒2) | 2 | 0.209 | 0.104 | 3.511 | 0.004 | 0.016 |
| Abbreviation: DF, degree of freedom; SS, sum of sqaures; MS, mean of squares | | | | | | | |

| **Table S6.** T test of plant, soil, paleoclimatic and current climatic variables (mean ± SE) between temperate and tropic-subtropical forests | | | | |  |
| --- | --- | --- | --- | --- | --- |
| Variable | | Temperate forest  (n = 100) | Tropic-subtropical forest (n = 140) | *t* | *p* |
| MAT | | 3.67 ± 0.68 | 18.19 ± 0.31 | -19.42 | <0.001 |
| MAP | | 654.36 ± 14.82 | 1701.37 ± 24.77 | -36.27 | <0.001 |
| MAT velocity | | 2.24 ± 0.15 | 1.22 ± 0.08 | 6.00 | <0.001 |
| MAP velocity | | 2.5 ± 0.21 | 1.01 ± 0.10 | 6.57 | <0.001 |
| MAT anomaly | | 7.64 ± 0.22 | 3.44 ± 0.08 | 17.88 | <0.001 |
| MAP anomaly | | 109.35 ± 2.68 | 46.14 ± 6.27 | 9.26 | <0.001 |
| Total carbon (C) | | 8.96 ± 0.42 | 5.79 ± 0.33 | 5.95 | <0.001 |
| Total nitrogen (N) | | 0.57 ± 0.02 | 0.42 ± 0.02 | 5.00 | <0.001 |
| Total phosphorus (P) | | 0.89 ± 0.03 | 0.44 ± 0.04 | 8.85 | <0.001 |
| N:P ratio | | 0.67 ± 0.03 | 1.55 ± 0.08 | -10.32 | <0.001 |
| C:N ratio | | 16.02 ± 0.46 | 14.04 ± 0.27 | 3.69 | <0.001 |
| C:P ratio | | 10.74 ± 0.6 | 23.02 ± 1.4 | -8.0882 | <0.001 |
| Total calcium (Ca) | | 11.04 ± 0.58 | 1.7 ± 0.22 | 14.96 | <0.001 |
| Total magnesium (Mg) | | 5.7 ± 0.23 | 2.68 ± 0.18 | 12.499 | <0.001 |
| pH | | 5.54 ± 0.07 | 4.51 ± 0.08 | 9.71 | <0.001 |
| PSD | | 2.59 ± 0.01 | 2.57 ± 0.00 | 2.88 | 0.00 |
| Total plant species richness | | 13.29 ± 0.89 | 37.93 ± 0.98 | -18.56 | <0.001 |
| Total plant basal area | | 16406.39 ± 937.98 | 14675.37 ± 508.79 | 1.62 | 0.11 |
| EM plant species richness | | 4.2 ± 0.17 | 3.95 ± 0.19 | 0.97 | 0.33 |
| Non-EM plant species richness | | 8.83 ± 0.76 | 31.89 ± 0.9 | -19.59 | <0.001 |
| EM plant basal area | | 13867.87 ± 986.38 | 5560.2 ± 397.17 | 7.81 | <0.001 |
| Non-EM plant basal area | | 2512.83 ± 302.77 | 8343.84 ± 330.08 | -13.02 | <0.001 |
| EM plant abundance | | 71.77 ± 6.9 | 27.57 ± 2.46 | 6.603 | <0.001 |
| Non-EM plant abundance | | 83.61 ± 8.36 | 212.82 ± 8.18 | -11.044 | <0.001 |

MAT, mean annual temperature; MAP, mean annual precipitation; PSD, particle size distribution; EM, ectomycorrhizal.
